# Supplementary material for: Fitness advantage of Bacteroides thetaiotaomicron capsular polysaccharide in the mouse gut depends on the resident microbiota
Source: eLife. 2023 Feb 9;12:e81212. doi: 10.7554/eLife.81212 (PMC10014078; doi:10.7554/eLife.81212)
Supplement: Supplementary file 1. — Whole-genome sequencings of all barcoded and untagged strains (WT and acapsular) were mapped against Bacteroides thetaiotaomicron strain VPI 5482 genome (CP092641.1) to identify genetics variants. Data are included in Figure 4—source data 1. [file elife-81212-supp1.docx]

**Supplementary File 1: Genomic variants (SNPs, small insertions, and deletions) in *B. theta* strains whole genome sequences**

| **Strain** | **Location** | **Ref.** | **Var.** | **Annotation** | **Annotation impact** | **Gene Name** | **Locus tag** | **Protein ID** | **Product** |
| --- | --- | --- | --- | --- | --- | --- | --- | --- | --- |
| *B. theta* acapsular untagged | CP092641.1:5398246 | A | C | Missense variant | MODERATE | MJ393_20770 | MJ393_20770 | UML60879.1 | pyridoxal phosphate-dependent aminotransferase |
| *B. theta* acapsular untagged | CP092641.1:763015 | T | A | Synonymous variant | LOW | pckA | MJ393_03275 | UML62109.1 | phosphoenolpyruvate carboxykinase (ATP) |
| *B. theta* acapsular tag 1 | CP092641.1:763015 | T | A | Synonymous variant | LOW | pckA | MJ393_03275 | UML62109.1 | phosphoenolpyruvate carboxykinase (ATP) |
| *B. theta* acapsular tag 1 | CP092641.1:5208844 | T | G | Upstream gene variant | MODIFIER | MJ393_19885 | MJ393_19885 | UML60714.1 | hypothetical protein |
| *B. theta* acapsular tag 1 | CP092641.1:5398246 | A | C | Missense variant | MODERATE | MJ393_20770 | MJ393_20770 | UML60879.1 | pyridoxal phosphate-dependent aminotransferase |
| *B. theta* acapsular tag 11 | CP092641.1:763015 | T | A | Synonymous variant | LOW | pckA | MJ393_03275 | UML62109.1 | phosphoenolpyruvate carboxykinase (ATP) |
| *B. theta* acapsular tag 11 | CP092641.1:5398246 | A | C | Missense variant | MODERATE | MJ393_20770 | MJ393_20770 | UML60879.1 | pyridoxal phosphate-dependent aminotransferase |
| *B. theta* acapsular tag 17 | CP092641.1:5398246 | A | C | Missense variant | MODERATE | MJ393_20770 | MJ393_20770 | UML60879.1 | pyridoxal phosphate-dependent aminotransferase |
| *B. theta* acapsular tag 17 | CP092641.1:763015 | T | A | Synonymous variant | LOW | pckA | MJ393_03275 | UML62109.1 | phosphoenolpyruvate carboxykinase (ATP) |
| *B. theta* acapsular tag 17 | CP092641.1:5208862 | A | G | Upstream gene variant | MODIFIER | MJ393_19885 | MJ393_19885 | UML60714.1 | hypothetical protein |
| *B. theta* acapsular tag 17 | CP092641.1:5208850 | A | AG | Upstream gene variant | MODIFIER | MJ393_19885 | MJ393_19885 | UML60714.1 | hypothetical protein |
| *B. theta* acapsular tag 17 | CP092641.1:5208844 | T | G | Upstream gene variant | MODIFIER | MJ393_19885 | MJ393_19885 | UML60714.1 | hypothetical protein |
| *B. theta* acapsular tag 17 | CP092641.1:5208867 | G | A | Upstream gene variant | MODIFIER | MJ393_19885 | MJ393_19885 | UML60714.1 | hypothetical protein |
| *B. theta* acapsular tag 19 | CP092641.1:763015 | T | A | Synonymous variant | LOW | pckA | MJ393_03275 | UML62109.1 | phosphoenolpyruvate carboxykinase (ATP) |
| *B. theta* acapsular tag 17 | CP092641.1:5398246 | A | C | Missense variant | MODERATE | MJ393_20770 | MJ393_20770 | UML60879.1 | pyridoxal phosphate-dependent aminotransferase |
| *B. theta* acapsular tag 2 | CP092641.1:763015 | T | A | Synonymous variant | LOW | pckA | MJ393_03275 | UML62109.1 | phosphoenolpyruvate carboxykinase (ATP) |
| *B. theta* acapsular tag 2 | CP092641.1:5208844 | T | G | Upstream gene variant | MODIFIER | MJ393_19885 | MJ393_19885 | UML60714.1 | hypothetical protein |
| *B. theta* acapsular tag 2 | CP092641.1:5398246 | A | C | Missense variant | MODERATE | MJ393_20770 | MJ393_20770 | UML60879.1 | pyridoxal phosphate-dependent aminotransferase |
| *B. theta* acapsular tag 21 | CP092641.1:763015 | T | A | Synonymous variant | LOW | pckA | MJ393_03275 | UML62109.1 | phosphoenolpyruvate carboxykinase (ATP) |
| *B. theta* acapsular tag 21 | CP092641.1:5398246 | A | C | Missense variant | MODERATE | MJ393_20770 | MJ393_20770 | UML60879.1 | pyridoxal phosphate-dependent aminotransferase |
| *B. theta* WT untagged | - | - | - | - | - | - | - | - | - |
| *B. theta* WT tag 1 | - | - | - | - | - | - | - | - | - |
| *B. theta* WT tag 11 | - | - | - | - | - | - | - | - | - |
| *B. theta* WT tag 17 | - | - | - | - | - | - | - | - | - |
| *B. theta* WT tag 19 | CP092641.1:2726474 | A | C | Missense variant | MODERATE | MJ393_09990 | MJ393_09990 | UML63380.1 | TonB-dependent receptor |
| *B. theta* WT tag 2 | - | - | - | - | - | - | - | - | - |
| *B. theta* WT tag 21 | - | - | - | - | - | - | - | - | - |
